# Rapid quantitative detection of *Klebsiella pneumoniae* in infants with severe infection disease by point-of-care immunochromatographic technique based on nanofluorescent microspheres

**DOI:** 10.3389/fbioe.2023.1144463

**Published:** 2023-02-08

**Authors:** Ying Chen, Lulu Sha, Wenqing Li, Liuyan Zhou, Bing Pei, Xinyu Bian, Yongxin Ji, Yiping Liu, Li Wang, Huan Yang

**Affiliations:** ^1^ School of Medical Technology, Xuzhou Medical University, Xuzhou, China; ^2^ Department of Clinical Laboratory, Suqian First People’s Hospital, Suqian, China; ^3^ Nanjing Nanoeast Biotech Co., Ltd., Nanjing, China; ^4^ Department of Clinical Laboratory, Xuzhou First People’s Hospital, Xuzhou, China

**Keywords:** PCR, immunochromatographic test strip (ICTS), POCT, strand exchange amplification (SEA), *Klebsiella pneumoniae*, rapid detection, nanofluorescent microsphere (nFM)

## Abstract

**Background:** *Klebsiella pneumoniae* (KP, *K. pneumoniae*) is one of the most important nosocomial pathogens that cause severe respiratory infections. As evolutionary high-toxic strains with drug resistance genes increase year by year, the infections caused by it are often accompanied by high mortality, which may be fatal to infants and can cause invasive infections in healthy adults. At present, the traditional clinical methods for detecting *K. pneumoniae* are cumbersome and time-consuming, and the accuracy and sensitivity are not high. In this study, nanofluorescent microsphere (nFM)-based immunochromatographic test strip (ICTS) quantitative testing platform were developed for point-of-care testing (POCT) method of *K. pneumoniae*.

**Methods:** 19 clinical samples of infants were collected, the genus-specific gene of *mdh* was screened from *K. pneumoniae.* Polymerase chain reaction (PCR) combined with nFM-ICTS based on magnetic purification assay (PCR-ICTS) and strand exchange amplification (SEA) combined with nFM-ICTS based on magnetic purification assay (SEA-ICTS) were developed for the quantitative detection of *K. pneumoniae.* The sensitivity and specificity of SEA-ICTS and PCR-ICTS were demonstrated by the existing used classical microbiological methods, the real-time fluorescent quantitative PCR (RTFQ-PCR) and PCR assay based on agarose gel electrophoresis (PCR-GE).

**Results:** Under optimum working conditions, the detection limits of PCR-GE, RTFQ-PCR, PCR-ICTS and SEA-ICTS are 7.7 × 10^−3^, 2.5 × 10^−6^, 7.7 × 10^−6^, 2.82 × 10^−7^ ng/μL, respectively. The SEA-ICTS and PCR-ICTS assays can quickly identify *K. pneumoniae*, and could specifically distinguish *K. pneumoniae* samples from non-*K. pneumoniae* samples. Experiments have shown a diagnostic agreement of 100% between immunochromatographic test strip methods and the traditional clinical methods on the detection of clinical samples. During the purification process, the Silicon coated magnetic nanoparticles (Si-MNPs) were used to removed false positive results effectively from the products, which showed of great screening ability. The SEA-ICTS method was developed based on PCR-ICTS, which is a more rapid (20 min), low-costed method compared with PCR-ICTS assay for the detection of *K. pneumoniae* in infants. Only need a cheap thermostatic water bath and takes a short detection time, this new method can potentially serve as an efficient point-of-care testing method for on-site detection of pathogens and disease outbreaks without fluorescent polymerase chain reaction instruments and professional technicians operation.

## 1 Introduction

*Klebsiella pneumoniae* (KP, *K. pneumoniae*) is one of the most harmful opportunistic bacteria and one of the main causes of nosocomial infections (Kong et al., 2018). It can cause bacterial pneumonia, liver abscesse (Chen et al., 2011; Nordmann and Poirel, 2014), sepsis and even other life-threatening consequences. In clinical investigations, drug-resistant *K. pneumoniae* was found to be highly contagious (Nordmann et al., 2012), especially in infants (Lee et al., 2020), because their immune system is fragile. The infections caused by *K. pneumoniae* are usually accompanied by high mortality (Arena et al., 2020), which could make severe challenges to clinical anti-infection treatment (Xu et al., 2017; Jia et al., 2022) and endanger infants’ health in developing countries (Zaidi et al., 2005; Li et al., 2021). At present, traditional microbial culture and drug resistance detection technologies consume a long time and are insensitive (Khan et al., 2014). Compared with the culture method, the molecular detection method is fast, sensitive and specific (Hooi et al., 2001). Among them, polymerase chain reaction (PCR) and immunochromatography are more commonly used. Traditional PCR detection methods based on agarose gel electrophoresis (PCR-GE) and real-time fluorescence quantitative PCR (RTFQ-PCR) (Chen et al., 2011; Nordmann and Poirel, 2014) have been widely used in the detection of pathogenic microorganism. However, PCR-GE can only be used for qualitative testing, and the following nucleic acid dyes in the agarose gel electrophoresis step are also toxic. RTFQ-PCR equipment is expensive (Glupczynski et al., 2016; Glupczynski et al., 2017; Song et al., 2020), which is not conducive to widespread clinical application.

**FIGURE 1:**
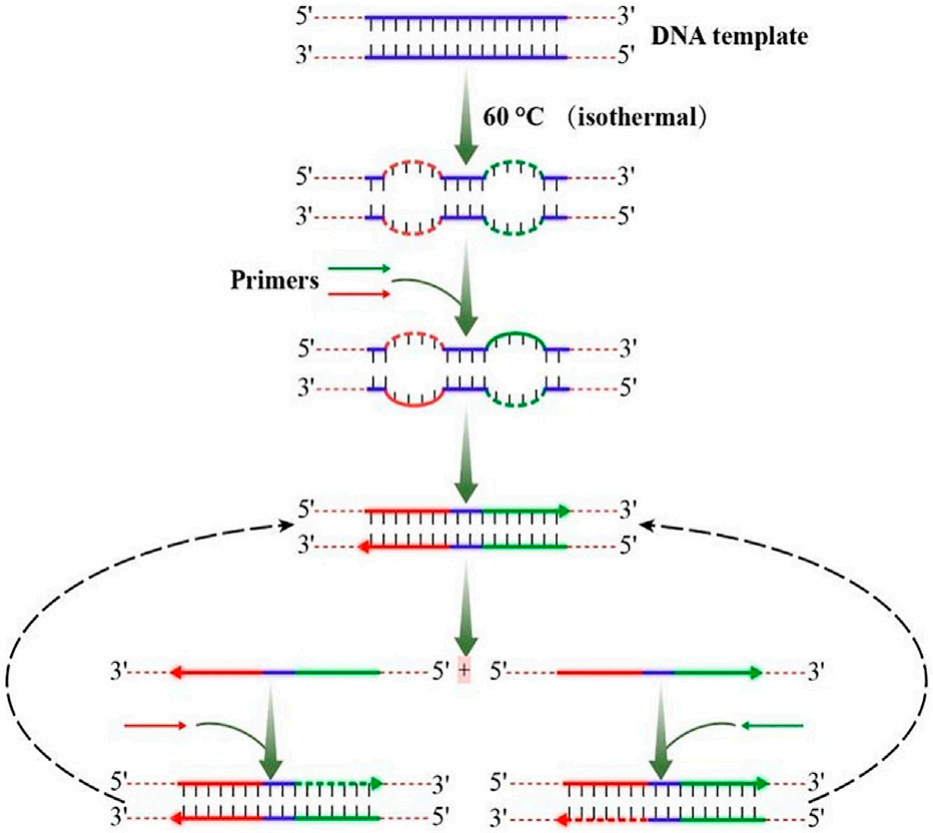
Schematic diagram of SEA.

In 2000, a study have developed a method, termed loop-mediated isothermal amplification (LAMP), that amplifies DNA under isothermal conditions (Notomi et al., 2000). This method employs a DNA polymerase and a set of four specially designed primers that recognize a total of six distinct sequences on the target DNA. Recently, a study using N.BspD6I nickel enzyme further explored the possible mechanism of DNA synthesis based on Bst DNA polymerase under non-template conditions (Zyrina et al., 2007). In 2016, strand exchange amplification (SEA) based on double-stranded DNA (dsDNA) degeneration bubbles was reported (Shi et al., 2016), which is the same with LAMP assay. However, the LAMP technology generally needs multiple pairs of primers and the primer design of LAMP is difficult, while SEA only uses one pair of primers. In SEA, a pair of primers (as shown in Figure 1) has been used to amplify DNA efficiently and steadily at a single temperature. As shown in the figure, a double-stranded DNA (dsDNA) exchanges one of its strands by a homologous single-stranded DNA (ssDNA) to form a heteroduplex product after completing the first primer expansion. DNA base have been added randomly by Bst DNA polymerase to the 3′ suspension due to its inherent nucleotide transferase activity. In the following rounds, more and more shift DNA strands with random 3′ suspension bases accumulate rapidly by the action of primers and Bst DNA polymerase. A new double strand can be formed if the newly synthesized strand has a 3′ drape DNA base, which complements the 3′ drape base of the previous replacement strand.

Immunochromatography test paper technology was first introduced in the 1980s. The design of immunochromatographic test strip (ICTS) can be currently used for pathogens’ nucleic acid detection, and is called “nucleic acid lateral flow immunoassay” (NALFIA) (Duong and Rhee, 2007; Chen et al., 2008; Chen et al., 2019; Zhuang et al., 2019; Chen et al., 2022). Immunochromatography test strip technology is currently widely used in fields requiring rapid antigen testing, especially in clinical medicine (Lin et al., 2022), agriculture (Dong et al., 2022; Lu et al., 2022), drug abuse (Suryoprabowo et al., 2021), food (Wang et al., 2022; Xing et al., 2022) and environmental semi-antigen pollutants (Ren et al., 2021), and microbial pollutants (Gao et al., 2021). In recent years, scientists have been working to develop ICTS methods for pathogen detection (Li et al., 2020a), but its low sensitivity has made it a bottleneck worldwide. With the advancement of nanotechnology, functional magnetic nanomaterials are gradually applied to biological detection due to their large specific surface area, superparamagnetism, and controllable size (Duong and Rhee, 2007; Chen et al., 2019). The magnetic beads enable the isolation or extraction of target molecule or substance due to the good biocompatibility and adequate functional groups for chemical fixation which have been applied to immunoassay. Silicon coated magnetic nanoparticles (Si-MNPs) we made can be used for specific bioaffinity capture of molecules. Moreover, nanofluorescent microsphere (nFM) have good thermal stability, dispersibility, biocompatibility, high fluorescence stability, surface modification, narrow particle size and high luminous efficiency, and nFM are rarely employed for the immunochromatography methods (Zhang et al., 2015; Chen et al., 2019; Zhuang et al., 2019; Chen et al., 2022; Chen et al., 2023).

In order to enable detection *in situ* or without expensive instrument conditions, we developed and validated a novel, rapid and sensitive immunoassay combined with strand exchange amplification (SEA) for isothermal and quantitative detection of *K. pneumoniae* in infants with severe infection disease by a point-of-care immunochromatographic technique based on nanofluorescent microspheres (SEA-ICTS) in this study. With the use of functional nanoparticles as signal amplifiers greatly increases the sensitivity of ICTS for fluorescence sensing, the entire process can be completed in 30 min and the results can be quantified by ICTS sensor reader without false positives or negatives.

The genus-specific gene of *mdh* (Sun et al., 2008; Thong et al., 2011) was screened from *K. pneumoniae* to investigate the application potential of new immune chromatography technology in early clinical detection as well as to compare the existing used classical microbiological methods, PCR-GE and the RTFQ-PCR method. The SEA-ICTS method was optimized based on PCR-ICTS we established in this study; However, the optimized SEA-ICTS method is a more rapid, low-costed method compared with PCR-ICTS assay for the detection of *Klebsiella pneumoniae* (KP, *K. pneumoniae*) in infants and can potentially serve as an efficient point-of-care testing (POCT) method for on-site detection of pathogens and disease outbreaks.

## 2 Material and methods

### 2.1 Bacterial culture and genomic DNA extraction

The bacterial strains used in this study included 19 clinical strains of *Klebsiella pneumoniae* (KP, *K. pneumoniae*) in infants collected from Nanjing Children’s Hospital (as shown in Table 1), one standard strain of *K. pneumonia* (ATCC 700603) and 14 clinical strains of non-*Klebsiella pneumoniae* (NKP) were provided from the Affiliated Hospital of Xuzhou Medical University and Xuzhou First People’s Hospital. Strains identification was performed by the traditional methods of bacteria culture and Vitek 2 Compact (BioMerieux, France), and all pathogenic strain DNA specimens are kept in the −80°C refrigerator. DNA extraction of all samples was carried out by TIANamp bacterial DNA kit (TIANGEN, Beijing, China) according to the manufacturer’s instructions. Ultraviolet spectrophotometer (Merinton SMA 1000, Beijing) was used to determine the ratio of A260/A280, and agarose electrophoresis was used to detect DNA integrity and evaluate DNA quality. The genome DNA (gDNA) of the above strain is stored at −80°C until it is thawed before analysis.

**TABLE 1:** Bacterial strains of infants used for SEA-ICTS and PCR-ICTS in this study.

No.	Sample	Age (months)	Sex	Disease	Species/strain
1	A12	1–5	F	Ventricular septal defect	*K. pneumoniae*
2	A17	1–5	M	Bronchopneumonia	*K. pneumoniae*
3	A18	1–5	M	Severe pneumonia	*K. pneumoniae*
4	A21	1–5	M	Septicopyemia	*K. pneumoniae*
5	A23	1–5	M	Bronchopneumonia	*K. pneumoniae*
6	A24	1–10 days	M	Neonatalpneumonia	*K. pneumoniae*
7	A28	1–5	M	Necrotizing enteritis	*K. pneumoniae*
8	A33	1–5	M	Bronchopneumonia	*K. pneumoniae*
9	A35	1–5	M	Neonatalpneumonia	*K. pneumoniae*
10	A37	1–10 days	M	Neonatalpneumonia	*K. pneumoniae*
11	A39	1–5	M	Bronchopneumonia	*K. pneumoniae*
12	A49	20–25 days	F	Sepsis	*K. pneumoniae*
13	A57	1–5	M	Bronchopneumonia	*K. pneumoniae*
14	A58	1–5	M	Pneumonia; sepsis	*K. pneumoniae*
15	A61	1–5	M	Bronchitis	*K. pneumoniae*
16	A62	1–5	M	Septicopyemia; severe pneumonia	*K. pneumoniae*
17	A65	1–5	M	Bronchopneumonia	*K. pneumoniae*
18	A66	1–5	M	Bronchitis; sepsis	*K. pneumoniae*
19	A127	6–10	M	Bronchopneumonia	*K. pneumoniae*

### 2.2 Reagents, chemicals and ICTS construction

All oligonucleotides, including target-specific primer sets (PCR and SEA) were synthesized by Sangon Biotech (Shanghai) Co., Ltd. (Shanghai, China). ICTS consists of six parts: sample pad, nitrocellulose membrane (NC), conjugate pad, absorption pad, polystyrene support card and detection area with test (T), and control line (C). Streptavidin-modified fluorescent microspheres were added into 1 mL of 0.01 mol/L PBS solution (pH 7.4) containing 0.9% Nacl, 1% BSA and 0.09% Proclin, then the mixture was centrifugated at 14,000 rpm for 15 min at 4°C. The supernatant was discarded and the residue was reconstituted in 1 ml of resuspension by vortex mixing and sonication. Repeat the above cleaning steps three times. Finally, 10 μL of Streptavidin-modified Fluorescent Microspheres were reconstituted in 100 μL of resuspension.

The sample pad was first treated with PBS solution (pH 7.4) containing 6% trehalose, 0.5% (v/v) Tween 20, 1% BSA, 0.5% pvpk30, 0.5% Tetronic 1,307 and 0.05% proclin300 followed by drying at 37°C for 10 h. The conjugate pad was saturated with Streptavidin-modified Fluorescent Microspheres and then dried at 37°C for 2 h. To obtain the detection area, Biotin−BSA and Anti-digoxin antibodies were diluted to the proper concentrations, filtered, and dispensed at the test line (T line) and control line (C line) respectively. The strip was assembled by laminating the sample pad, conjugate pad, NC membrane, and absorbent pad onto a backing card. The conjugate pad and the absorbent pad were separately attached to the ends of the NC membrane with overlaps of 2 mm. The sample pad was pasted onto the conjugate pad with a 2 mm overlap. The assembled backing was cut into the width of 4 mm and placed in a sealed bag with desiccant. In the end, it should be kept away from light and stored at 4°C.

### 2.3 Establish and optimize the nucleic acid amplification reaction conditions of SEA and PCR

#### 2.3.1 Design of SEA and PCR primers

The genus-specific gene of *mdh* sequence of *Klebsiella pneumoniae* strain was obtained from GenBank nucleotide sequence database (http://www.ncbi.nlm.nih.gov). The specific primers for SEA (sF and sR) and PCR (F and R) were designed by NUPACK network software and Primer Premier 5.0 (Premier Biosoft International, Palo Alto, CA). Forward primer of sF and F were labeled with biotin at the 5′ end and reverse primer of sR and R were labeled with digoxigenin at the 3′ end. The primers above were synthesized by Sangon Biotech (Shanghai) Co., Ltd.

#### 2.3.2 Establish and optimize of SEA and PCR

The SEA reaction was carried out within the final volume of 25 μL reaction system containing 7.5 μL primer, 2.5 μL DNA template, 4 μL buffer B of DNA polymerase and 11 μL buffer A of isothermal amplification buffer (Qingdao Naide Biotechnology Co., Ltd.) to form a reaction system, blow and mix well, and briefly centrifuge to remove bubbles. The product is visualized under ultraviolet light through 3% agarose gel electrophoresis. SEA’s identification and experimental procedures are auxiliary information. In order to optimize the reaction conditions, the fluorescence values of *K. pneumoniae* (ATCC 700603) genomic DNA (gDNA) were measured in a constant temperature of water bath of 60°C, 61°C, 62°C, 63°C, 64°C and 65°C. Each SEA reaction contains a positive and negative control, and all SEA experiments are repeated.

The PCR reaction was performed in a final volume of 25 µL containing 12.5 μL green *Taq* mixture (Vazyme, Nanjing), 1 μL forward primer (10 μmol/L), 1 μL reverse primer (10 μmol/L), 1 μL sample DNA and sterile deionized water. The PCR reaction was carried out on a Bio-Rad PCR system under the following conditions: 95°C 5 min, 30 cycles of 95°C 30 s, 58.5°C 30 s and 72°C 30 s, finally extend for 7 min at 72°C. The products were visualized under ultraviolet light through 1.5% (w/v) agarose gel electrophoresis (PCR-GE) to minimize the possibility of non-specific amplification.

### 2.4 Purification SEA/PCR products

**FIGURE 2:**
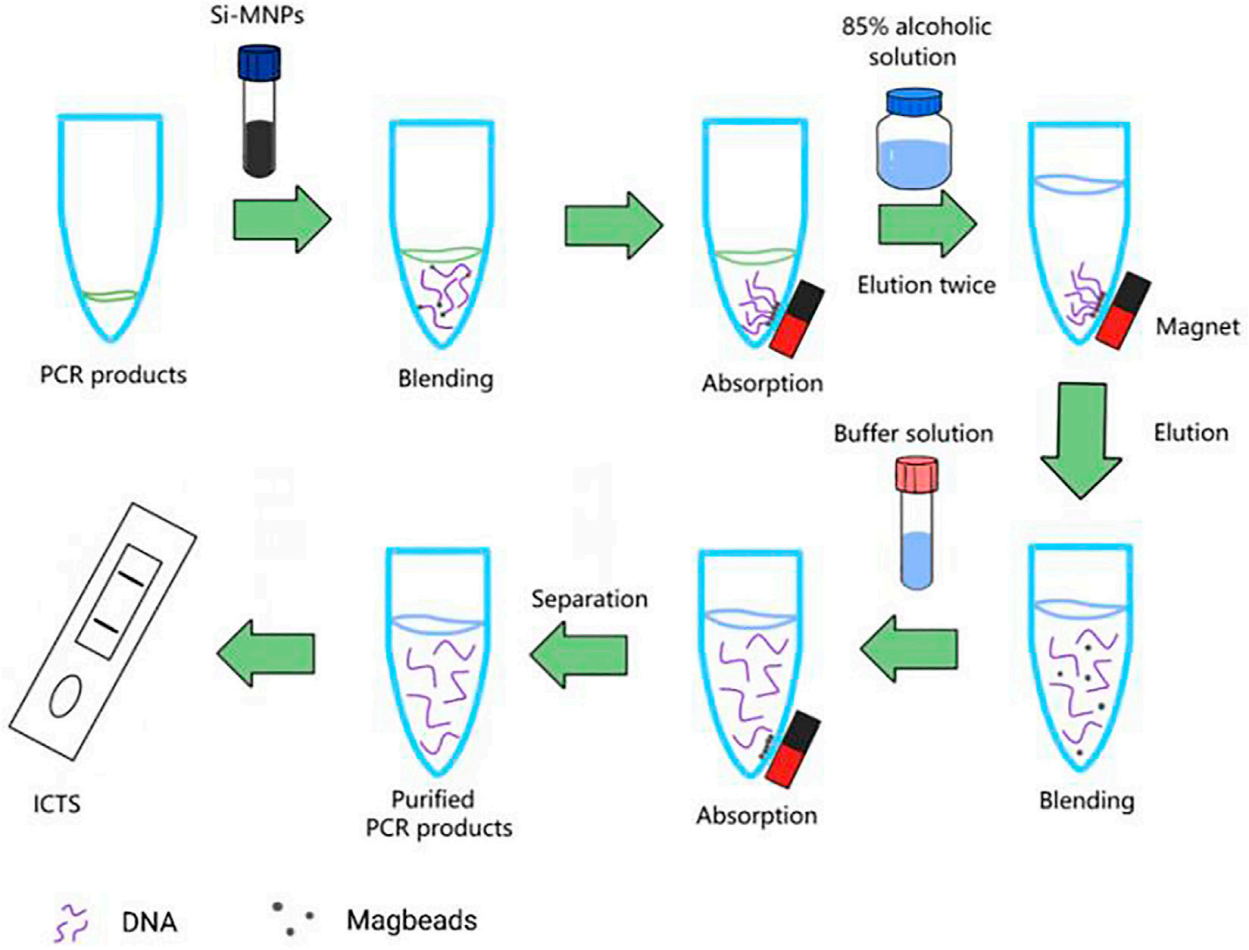
The illustration of Si-MNPs purified SEA/PCR products.

Add 5 μL SEA or PCR products and 9 μL Si-MNPs, the mixture was hatched at room temperature (RT), and slowly mix for 5 min to fully adsorb DNA fragments. The Si-MNPs-DNA complex was then fixed with magnets for 2 min and washed twice with alcohol solution (85%). Suspend Si-MNPs with 15 μL of sterile deionized water, and then remove Si-MNPs from the nucleic acid products. Then the purified SEA or PCR products can be used for ICTS detection or stored at −20°C. The whole process is shown in Figure 2.

### 2.5 Establish SEA-ICTS and PCR-ICTS for the detection of *Klebsiella pneumoniae*

Add the reaction mixture of 100 μL containing purified 15 μL SEA or PCR products and 85 μL PBS (pH 7.4) to the reaction tube. Then, the mixed solution were directly added into our ICTS sample pad, which was the specific positions designated as the capture test lines on the strip, and 100 μL PBS solution was used as the blank control. The fluorescence value was read by ICTS sensor reader of Nanoeasy 1700 (Nanoeast, Nanjing, China) which we made after 2 min and the relevant image was recorded by fluorescence imager. The quantification of SEA or PCR products can be completed in 3 min.

### 2.6 Detection of sensitivity and specificity of SEA-ICTS and PCR-ICTS

The genus-specific gene of *mdh* was used to detect the specificity of microorganisms of *K. pneumoniae,* and other pathogenic samples (as shown in Table 1) of SEA-ICTS and PCR-ICTS methods as well as to evaluate the specificity of the test paper. The SEA amplification were carried out using 28.2 to 2.82 × 10^−8^ ng/μL standard DNA to evaluate the detection limits of SEA-GE and SEA-ICTS (Figure 7). The PCR amplification were carried out with standard DNA of 7.7 to 7.7 × 10^−7^ ng/μL to evaluate the detection limits of PCR-GE and PCR-ICTS (Figure 7). Sterile deionized water is used as a negative control, and the concentration is repeated three times per concentration. The ICTS sensor reader is used for detection. In the fluorescence threshold test, the signal value is defined as 500 (detection limit). If the signal value is greater than 500, the result is positive, otherwise the signal value is less than 500, which is negative.

*K. pneumoniae* standard strain (ATCC 700603), 19 clinical isolates and 14 non-*Klebsiella pneumoniae* reference strains were used to evaluate the specificity of SEA-ICTS and PCR-ICTS assays (as shown in Table 1). And sterile phosphate-buffered saline (PBS buffer, pH 7.4) was used as a negative control for DNA extraction. The reaction was carried out under optimized conditions. All samples were performed in duplicate.

### 2.7 RTFQ-PCR quantitative detection

RTFQ-PCR reaction was carried out on the StepOne PULS fluorescence quantitative PCR analyzer (ABI, Foster, CA, United States) using a 2X SG Fast qPCR Master Mix (High Rox, BBI, ABI). Each 20 μL reaction mixture contains 10 μL 2X SybrGreen qPCR main mixture (High Rox, B639273, BBI, ABI), 0.4 μL primer F (10 μmol/L), 0.4 μL primer R (10 μmol/L), 2 μL DNA template and sterile deionized water with a volume of up to 20 μL. The cyclic conditions are as follows: 95°C 3 min, 45 cycles of 95°C 5 s, 60°C 30 s. The sensitivity of RTFQ-PCR (*mdh*) was evaluated using gDNA (1.74 × 10^7^–1.74 × 10^3^ copies/μL) of different concentrations of *K. pneumoniae* strains. All processes were repeated three times, including negative and positive control.

## 3 Results

### 3.1 The principle of ICTS quantitative testing platform based on nanofluorescent microsphere (nFM)

**FIGURE 3:**
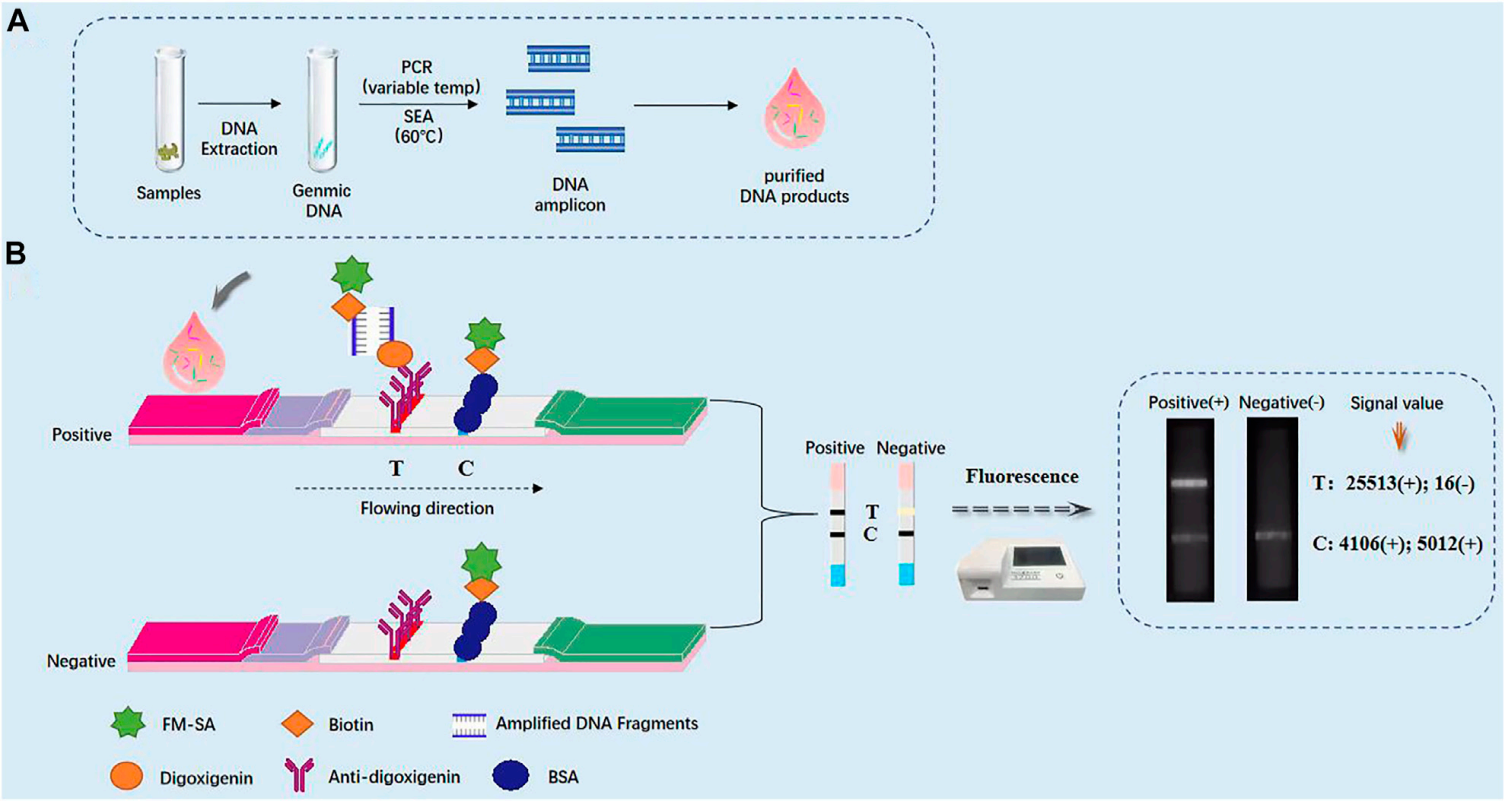
Schematic diagram of immunochromatographic test strip (ICTS). **(A)** Schematic diagram of immunochromatographic test strip for the detection of *Klebsiella pneumoniae*. **(B)** Photograph of the packaged ICTS and its internal structure, consisting of a sample pad, a conjugate pad, a detection region with test and control lines, and an absorbent pad.

Genomic DNA is extracted, and then performed to nucleic acid isothermal amplification of SEA and variable temperature amplification of PCR, respectively (Figure 3). The specific primers of genus-specific gene *mdh* for SEA (sF and sR) and PCR (F and R) were labelled on 5′-end with biotin and digoxigenin, respectively. After purification of SEA or PCR products, the reaction mixture was directly loaded on the immunochromatographic test strips based on nFM. One end of the enlarged products is marked with biotin and bound to FM-labeled streptavidin, and the other end is marked with digoxin, which binds to the anti-digoxin antibody on the test line (T), redundant streptavidin-coated nFM can be combined with biotin-labeled BSA on the control line (C), thereby a portable test paper reader of fluorescence detection system can receive fluorescence signal. Using the ICTS sensor reader to detect stronger fluorescence signals, and the results can be quantified and shared in real time through the network. In the presence of the target, the positive result appeared with two data at the T and C line positions on the strip, while in the absence of the target, the negative result with a very low value (value < 500) at the T line position on the strip. Negative control using PBS instead of SEA or PCR products did not result in any value on the T lines. So the SEA-ICTS and PCR-ICTS methods can be used to rapid and specific analysis of gDNA isolated from *K. pneumoniae.* No false-positive or false-negative results were ever seen, and the results were comparable to PCR-GE and RTFQ-PCR (as shown in Table 1).

### 3.2 Characterization of nanofluorescent microspheres-streptavidin (nFM-SA)

**FIGURE 4:**
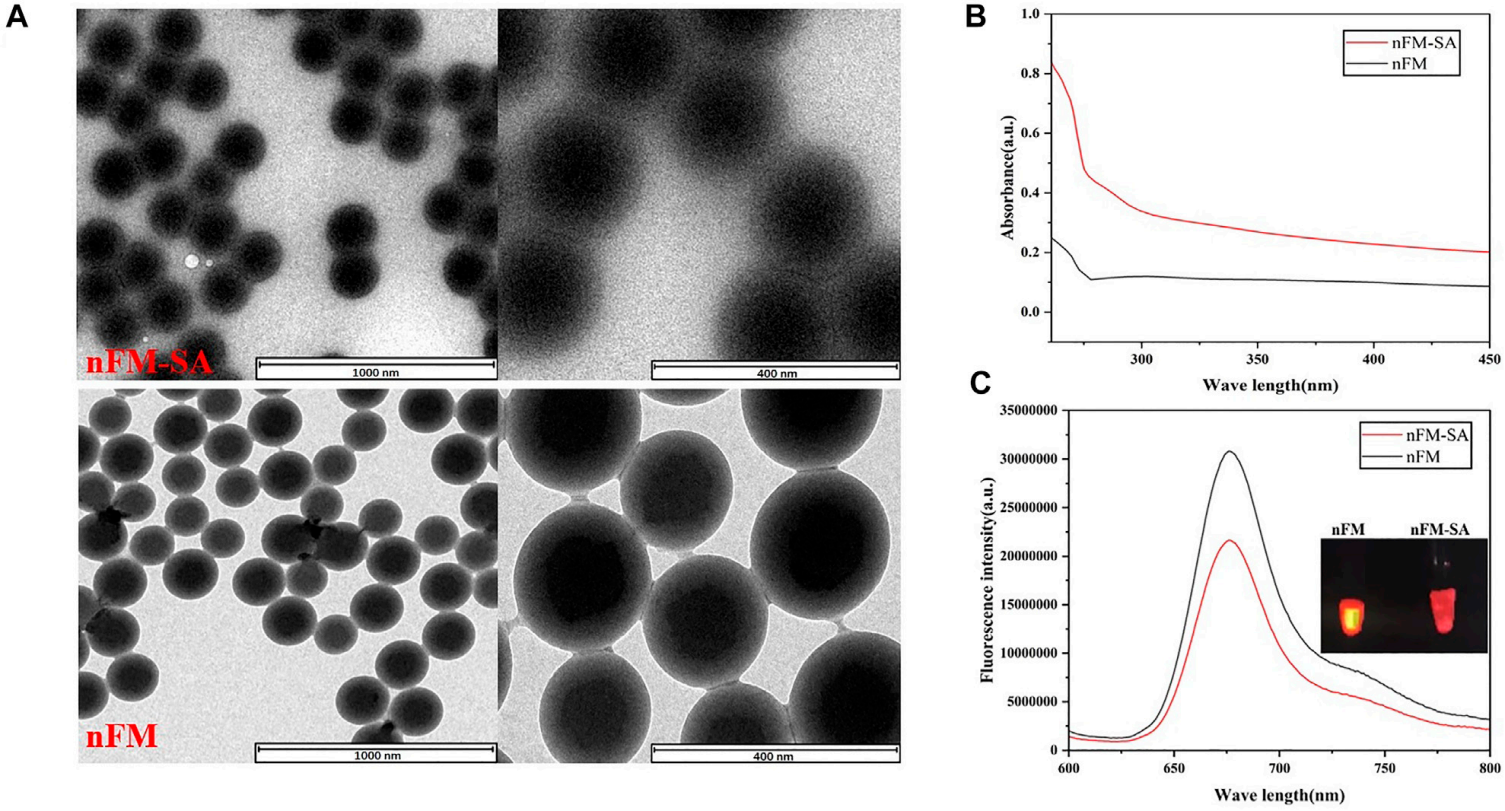
Characterization of nFM and nFM-SA conjugates. **(A)** Transmission electron microscopy (TEM) images of synthesized nFM and nFM-SA conjugates, **(B)** Ultraviolet visible (UV–vis) absorption, and **(C)** fluorescence emission spectra of free nFM and nFM-SA conjugates.

The nanofluorescent microspheres-streptavidin (nFM-SA) conjugates was prepared based on coupling method and the corresponding characterization was performed. Figure 4A shows that the nFM have a standard spherical structure before and after SA modification. From the transmission electron microscopy image, compared with free-nFM, the surface of the nFM-SA microspheres was covered with a membrane-like structure. Both nFM and nFM-SA have good uniformity and dispersibility and are soluble in water and other common solvents. To confirm the success of cross-linking, fluorescence spectra and ultraviolet visible (UV-Vis) absorption were used. Compared with unmodified nFM, nFM-SA has obvious a protein characteristic peak near 280 nm in UV–visible absorption spectra, indicating that SA has successfully combined with nFM (Figure 4B). As shown the fluorescence spectroscopy in Figure 4C, the maximum emission wavelength of nFM is 676 nm, and the fluorescence intensity of nFM-SA decreased compared with that of the unmodified nFM. The main reason is attributed to the fact that the organic structure of SA wrapped on the surface of the fluorescent microspheres absorbs part of the fluorescence of the nFM (Shang et al., 2021). These data indicate that the nFM-SA complex were successfully prepared and fully met the test strip requirements.

### 3.3 Verification of the SEA and PCR technique for nucleic acid detection

**FIGURE 5:**
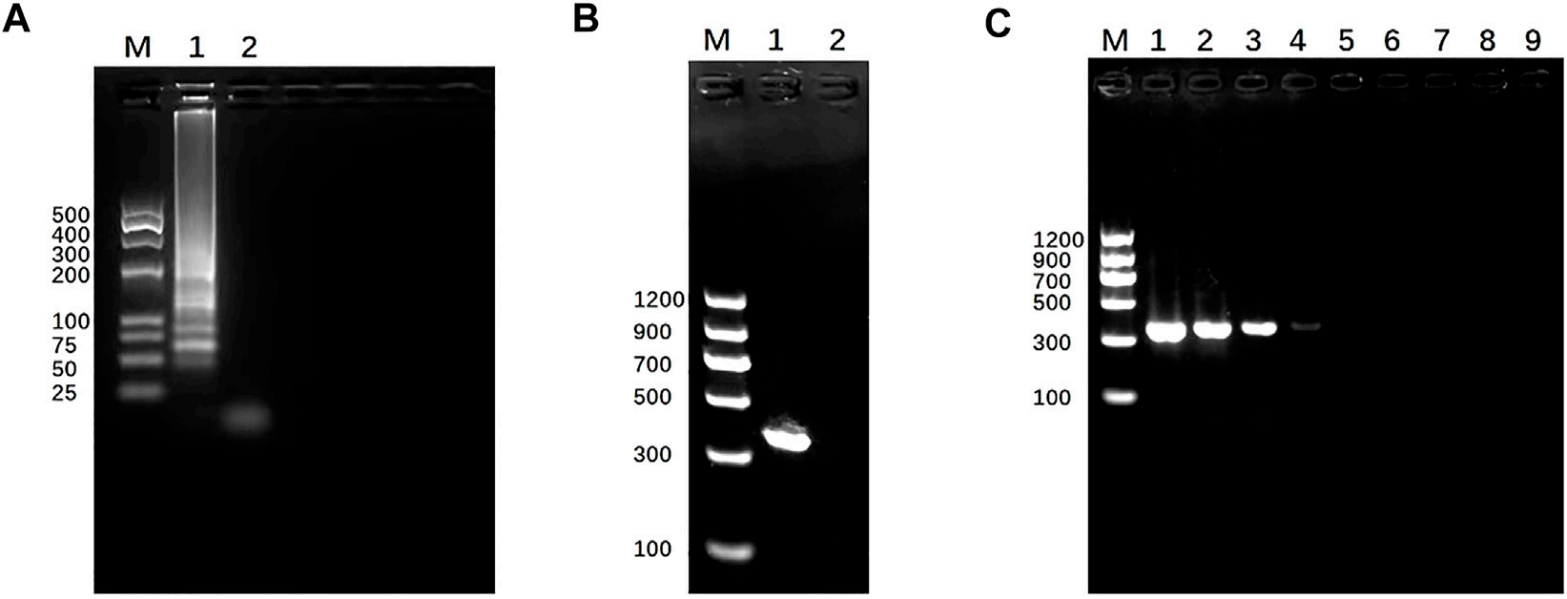
Establishment and identification of SEA and PCR. **(A)** SEA result. Lane M, DL 500 DNA marker. Lane 1, SEA product of *mdh* for *Klebsiella pneumoniae* by 3% (w/v) agarose gel electrophoresis. Lane 2, negative control. **(B)** PCR result. Lane M, DL 2000 DNA marker. Lane 1, PCR products of *mdh* for *Klebsiella pneumoniae* determined by 1.5% (w/v) agarose gel electrophoresis. Lane 2, negative control.**(C)** Detection sensitivity of PCR-GE using serially diluted *Klebsiella pneumoniae* (*mdh*); lane M, DL1200 marker; lanes 1–8, DNA concentration from 7.7 to 7.7 × 10^−7^ ng/μL. Lane 9, negative control. The size of the PCR products (*mdh*) was 364 bp.

To test the feasibility of the SEA-ICTS and PCR-ICTS platform for nucleic acid detection, a genus-specific gene of *mdh* for *K. pneumoniae* was selected. The SEA and PCR products were visualized under ultraviolet light through agarose gel electrophoresis (GE), and compared to DNA Marker (TaKaRa, Dalian, China) to verify the products or the size of amplicons in order to minimize the possibility of non-specific amplification. The results showed that the PCR and SEA products were successfully amplified, while negative control did not show any amplification results (Figure 5).

### 3.4 The results of before and after purification of SEA/PCR products

In order to correctly detect SEA or PCR products, Si-MNPs were used as a purification and signal amplifier to remove false positive results. As shown in Table 2, the test line value in the dilution group (blank control) showed that the background signal value of the fluorescence intensity is relatively low, indicating that the test strip itself does not produce false positive. In the negative control groups, the fluorescence intensity of the pre-purification signal of the negative samples were 5,643 and 4,548, indicating that the amplification of the non-target band caused false positive results. The signal intensity after purification were reduced to 176 and 187, indicating that most non-target strips have been cleared during purification, and ICTS can be used to detect *K. pneumoniae* as an effective tool. There is no significant difference in signal values between S1, S2 and S3 positive samples before and after purification, which are similar to the results of P1, P2 and P3. These results are consistent with the results of agarose gel electrophoresis. Therefore, the ICTS strips had good repeatability and strong screening ability, which can eliminate false positive results.

**TABLE 2:** The fluorescence intensity results of before and after purification of SEA/PCR products.

Variable	SEA product (before purification)		SEA product (after purification)	
	T line	C line	T line	C line
Diluent	53	6,978		
Negative control	2,590	5,643	176	6,523
S1	47,368	5,319	50,483	6,409
S2	44,648	6,214	47,521	5,432
S3	43,211	5,980	43,790	4,320
Variable	PCR product (before purification)		PCR product (after purification)	
	T line	C line	T line	C line
Diluent	46	7,223		
Negative control	2,312	4,548	187	4,381
P1	35,604	4,727	26,936	4,709
P2	29,396	6,465	22,720	4,392
P3	36,292	5,743	29,874	4,507

S1, S2, S3 represent SEA amplified products of *mdh* gene. P1, P2, P3 represent PCR amplified products of *mdh* gene.

### 3.5 Optimize the reaction conditions of SEA-ICTS

**FIGURE 6:**
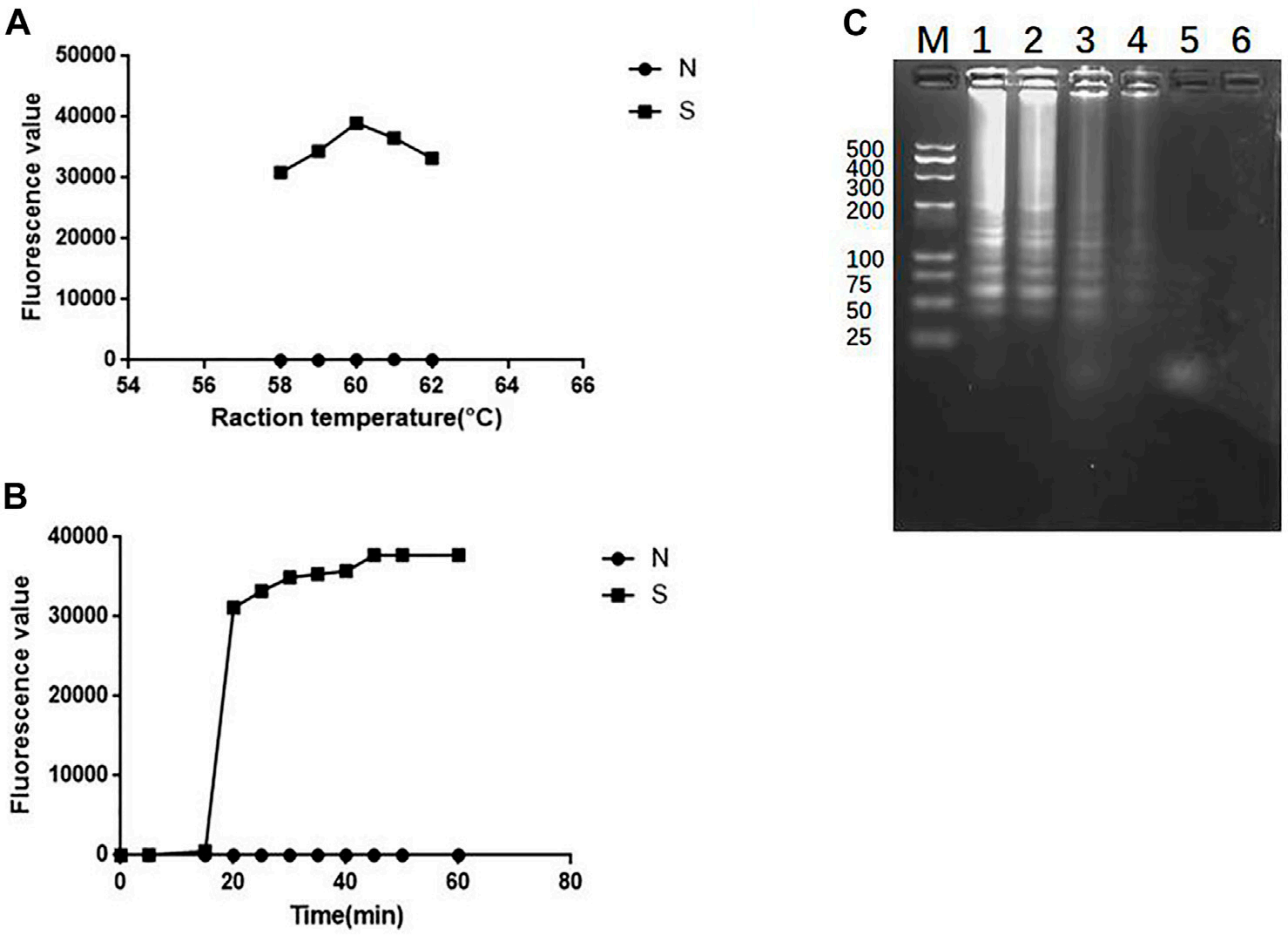
Optimize the reaction conditions of SEA. **(A)** Fluorescence values of standard strain of *Klebsiella pneumonia* at 58°C, 59°C, 60°C, 61°C and 62°C, respectively by SEA-ICTS; **(B)** Fluorescence values of parallel samples collected by SEA-ICTS every 5 min from 0 to 60 min at 60°C; **(C)** SEA product determined by 3% agarose gel electrophoresis. Lane M, DL 500 DNA marker. Lane 1, 35 min. Lane 2, 30 min. Lane 3, 25 min. Lane 4, 20 min. Lane 5, 15 min, Lane 6, negative control.

In order to optimize the reaction conditions of temperature and time, the standard strain of *K. pneumoniae* was used as a target to optimize the reaction temperature in a constant temperature of water bath of 58°C, 59°C, 60°C, 61°C and 62°C. All SEA-ICTS experiments were repeated and contain negative control. As shown in Figure 6A, the optimum reaction temperature was 60°C. Then, the fluorescence values of SEA amplicons were collected every 5 min from 0 to 60 min under 60°C as the optimize the reaction temperature. As shown in Figure 6B, when the reaction time reached 20 min, the fluorescence values was 31,153, which was significantly different from the negative control. The SEA products were also visualized by agarose gel electrophoresis (SEA-GE), the results were consistent with SEA-ICTS (Figure 6C). Therefore, 60°C reaction for 20 min was determined as the optimal reaction conditions for SEA-ICTS assay.

### 3.6 Detection of sensitivity and of SEA-ICTS and PCR-ICTS

**FIGURE 7:**
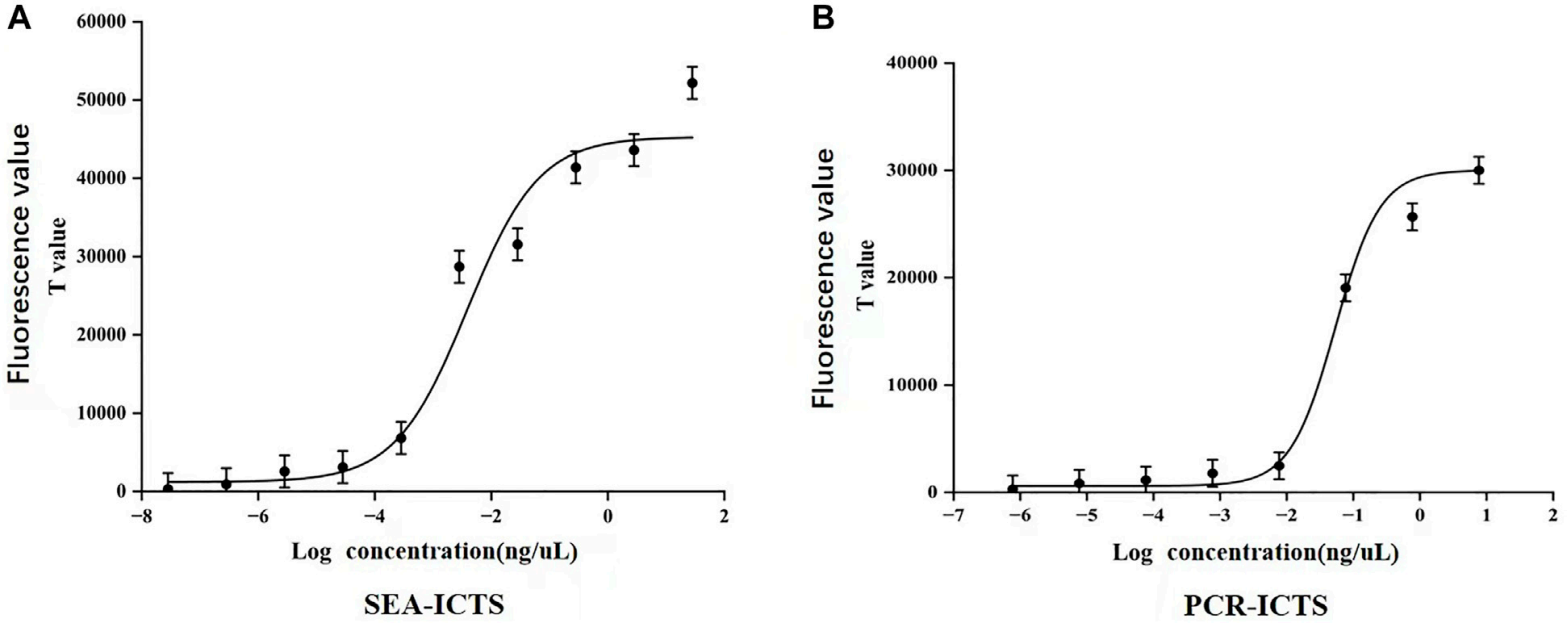
Sensitivity detection of *Klebsiella pneumoniae*. **(A,B)**, The correlation curves were established with the logarithmic value of DNA concentration as the abscissa and the signal value (T value) of ICTS as the ordinate. Each value was derived from three independent experiments, and the error bars mean standard deviations.

In SEA-ICTS method, the *mdh* gene was detected by using a concentration of 28.2 ng/μL and ten times continuously diluted. The results determine the sensitivity of SEA-ICTS. In SEA-ICTS, the fluorescence signal values of standard DNA dilution range from 51686.88 to 310.07, and the concentration of *mdh* decreased from 28.2 to 2.82 × 10^−8^ ng/μL. The relationship between different concentrations and the test value was observed {Y = 45286.1 − 44072.9/(1 + exp^[(x+2.4)/0.6])}, the correlation analysis shows that the Pearson correlation coefficient (Pearson’s r) between DNA concentration and ICTS results is R^2^ = 0.9396 (Figure 7A). The results showed that the sensitivity of SEA-ICTS for the detection of *mdh* was 2.82 × 10^−7^ ng/μL.

In PCR-ICTS method, the *mdh* gene was detected by using a standard strain concentration of 7.7 ng/μL and diluted ten times continuously. The results determine the sensitivity of PCR-GE and PCR-ICTS (Figure 5C, 7). In PCR-ICTS, the fluorescence signal values of standard DNA dilution range from 286.52 to 30,462. The relationship between different concentrations and the test value was observed {Y = 30008.6 − 29388/(1 + exp^[(x + 1.3)/0.3])}, and *mdh* concentration decreased from 7.7 to 7 × 10^−7^ng/μL. Correlation analysis shows that the Pearson correlation coefficient (Pearson’s r) between DNA concentration and ICTS results is R^2^ = 0.9411. The results show that the detection limits of the *mdh* gene used by PCR-ICTS is 7.7 × 10^−6^ ng/μL, which is 1,000 times that measured by PCR-GE of 7.7 × 10^−3^ ng/μL (Figure 5C). However, the detection limits of the *mdh* gene used by SEA-ICTS is 2.82 × 10^−7^ ng/μL, which is 10 times that measured by PCR-ICTS.

### 3.7 Specificity detection of clinical sample by SEA-ICTS and PCR-ICTS

**FIGURE 8:**
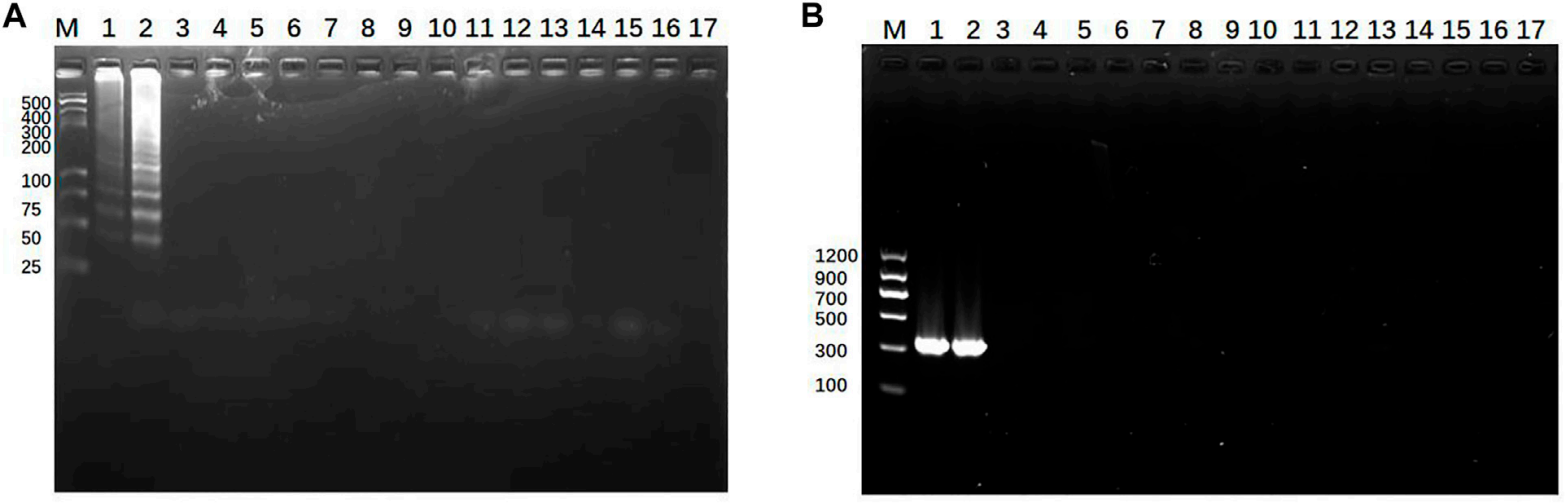
Specificity detection of *Klebsiella pneumoniae*. **(A)** Detection specificity of SEA-GE using *Klebsiella pneumoniae* (*mdh*) and non-*Klebsiella pneumoniae*: lane M, DL 500 DNA marker; lanes 1-2, *Klebsiella pneumoniae* with different DNA concentrations; lanes 3–16, non-*Klebsiella pneumoniae,* Lane 17, negative control. **(B)** Detection specificity of PCR-GE using *Klebsiella pneumoniae* and non-*Klebsiella pneumoniae*: lane M, DL 1200 DNA marker; lanes 1-2, *Klebsiella pneumoniae*; lanes 3–16, non-*Klebsiella pneumoniae*. Lane 17, negative control.

To verify the specificity of new methods, *Klebsiella pneumoniae* (KP, *K. pneumoniae*) specimens from infants and 14 cases of non-*Klebsiella pneumoniae* (nKP) biopathogens were tested. The results showed that only *K. pneumoniae* strains showed characteristic amplification band, while nKP strains and negative control did not show any amplicon (Figure 8). Then, 19 cases *Klebsiella pneumoniae* (KP, *K. pneumoniae*) specimens from infants and 14 cases of non-*Klebsiella pneumoniae* (nKP) biopathogens were tested by SEA-ICTS and PCR-ICTS. The results showed that the detection of KP and nKP with ICTS technology were consistent with traditional detection methods, which showed a high degree of specificity (Table 3).

**TABLE 3:** Comparison of SEA-ICTS and PCR-ICTS results with PCR-GE and Vitek 2 compact results (*mdh*).

NO.	Sample type and species	NAA-ICTS		NAA-GE		Vitek 2 compact
		SEA-ICTS	PCR-ICTS	SEA-GE	PCR-GE	
1	*Klebsiella pneumoniae* (ATCC 700603)	+	+	+	+	+
2–20	*Klebsiella pneumoniae* (19 clinical isolates of infants)	+	+	+	+	+
21	*E. faecium*	−	−	−	−	−
22	*S. mitis*	−	−	−	−	−
23	*S. pneumoniae*	−	−	−	−	−
24	*S. epidermidis*	−	−	−	−	−
25	*S. aureus*	−	−	−	−	−
26	*A. baumannii*	−	−	−	−	−
27	*A. cloacae*	−	−	−	−	−
28	*E. coli*	−	−	−	−	−
29	*P. Aeruginosa*	−	−	−	−	−
30	*S.maltophilia*	−	−	−	−	−
31	*S. boydii*	−	−	−	−	−
32	*Y. enterocolitia*	−	−	−	−	−
33	*Salmonella*	−	−	−	−	−
34	*C. albicans*	−	−	−	−	−

NAA, nucleic acid amplification.

### 3.8 RTFQ-PCR quantitative detection

**FIGURE 9:**
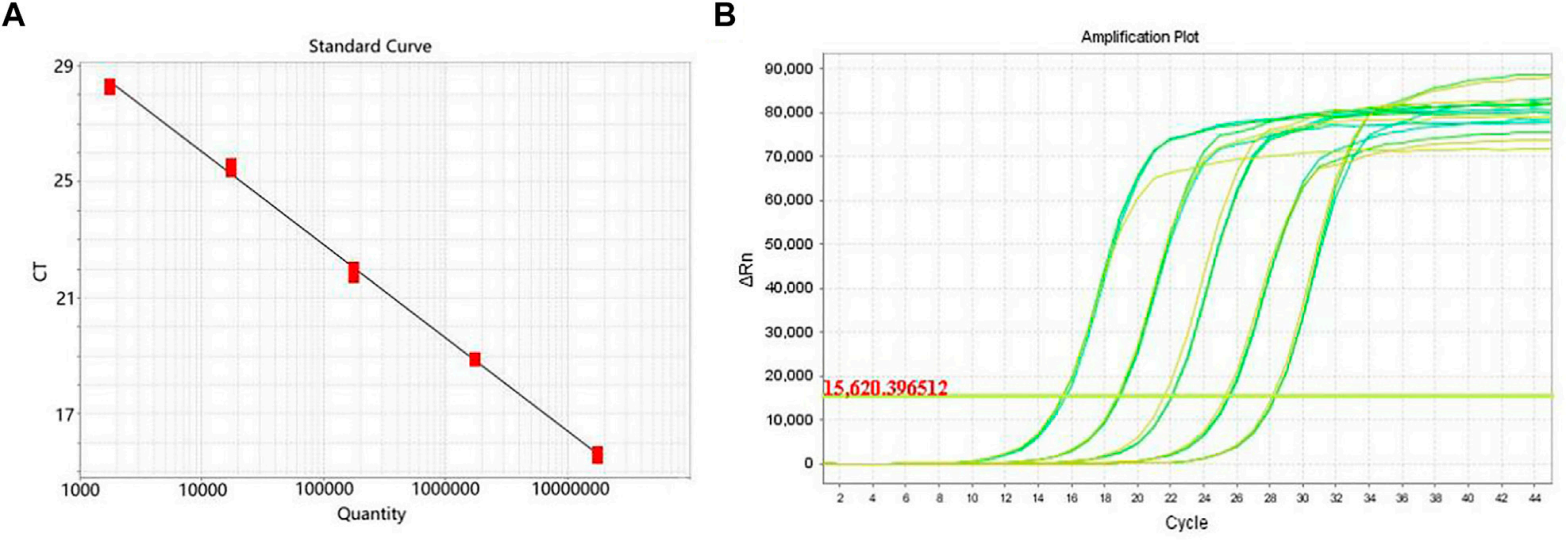
Detection sensitivity of RTFQ-PCR using standard strain of *Klebsiella pneumoniae*. **(A)** The standard curve of the RTFQ-PCR for the detection of 5 *Klebsiella pneumoniae* DNA samples from 5.8 × 10^−2^ to 5.8 × 10^−6^ ng/μL; **(B)** The amplification curves of RTFQ-PCR method of these 5 *Klebsiella pneumoniae* samples with different concentrations. Numbers 1–5 represent the different concentrations.

The genus-specific gene of *mdh* of *K. pneumoniae* was detected by the gold standard detection method of RTFQ-PCR. According to Figure 9A, there is a good linear relationship between the logarithm of DNA concentration and the periodic threshold (Ct) value of RTFQ-PCR. The linear equation is y = −3.211x + 38.869, and the R^2^ value exceeds 0.99. The minimum detection limit for plasmids containing the target gene is 67.3 copies/uL, which is converted to a DNA concentration with the sensitivity of 2.5 × 10^−6^ ng/uL. From Figure 9B, it can be judged that the detection results of different DNA concentrations of *K. pneumoniae* are very repetitive and sensitive.

## 4 Discussion

In recent years, due to long-term excessive and improper clinical use of carbapenem antibiotic etc., the detection rate of *K. pneumoniae* has been increasing, especially in hospital-born infants (Yu et al., 2016) with serious infectious diseases (Lôme et al., 2018; Porreca et al., 2018; Huang et al., 2020).

In this study, 19 samples used SEA-ICTS, PCR-ICTS, qualitative testing of agarose gel electrophoresis, and traditional clinical methods as well as the molecular gold standard technology of RTFQ-PCR for the detection of the genus-specific gene of *K. pneumoniae* from infants.

Under optimum working conditions, the detection limits of PCR-GE, RTFQ-PCR, PCR-ICTS and SEA-ICTS are 7.7 × 10^−3^, 2.5 × 10^−6^, 7.7 × 10^−6^, 2.82 × 10^−7^ ng/μL, respectively. The PCR-ICTS and the SEA-ICTS assays have detected *K. pneumoniae*, and could specifically distinguish *K. pneumoniae* samples from non-*Klebsiella pneumoniae* samples. Moreover, the fluorescence intensity by ICTS was higher than electrophoretic band in agarose gel electrophoresis detection. The results showed that SEA-ICTS and PCR-ICTS detection platform in this study have high sensitivity and strong screening ability, which can eliminate false negative results.

Immune chromatography can only detect specific bacterial antibodies based on monoclonal or polyclonal (Li et al., 2020b), and most test strips are only used for qualitative experiments. In this study, streptavidin-modified nFM were used to capture amplification products labeled with biotin. Compared with colloidal gold, nFM has higher signal values, a wider linear range, better interoperability and repeatability, and is also suitable for quantitative detection. In addition, the high degree of specific affinity and multi-stage amplification between biotin and nFM can be used to improve the sensitivity of detection lines to immune binding and trace analysis.

In recent years, new ICTS-based detection methods have emerged, which means that ICTS methods are practical in many fields. These methods reduce the detection workload and the possibility of bacterial contamination (Kuo et al., 2015; Vyas et al., 2015). The ICTS method, combined with some special equipment, can process large-volume samples in complex analytical processes. For example, Xu et al. (2013) proposed a new information and communication technology that uses multiclonal antibodies to label FMs to detect *C. jejuni*, which can only qualitatively detect *clostridium difficile*. Zhang et al. (2013) developed a plant pathogens (Cmn and Pss) detection method based on microsphere-based fluorescent immunoassay, which analysis time (1 h) was much shorter compared with ELISA (6–8 h). However, flow cytometry and more samples are required in this method. Blavikova et al. (2011) developed an ICTS detection method requiring special equipment and complex analytical processes (total analysis time is 16 h).

However, the ICTS quantitative testing platform developed in this study have advantages such as being simple to operate, low-cost, high sensitivity and specificity as well as rapid quantitative detection. It can achieve quantitative detection of target DNA sequences through low-costed and small-sized detection equipment. After purification process of magnetic beads, the false positive can also be reduced; Therefore, the ICTS quantitative testing platform are popularized and used in areas with poor clinical and medical equipment where vulnerable to *K. pneumoniae* pathogens. In contrast, traditional microbial methods usually take 2–3 days to obtain qualitative results, which is time-consuming and laborious. PCR assay based on agarose gel electrophoresis (PCR-GE) takes a relatively long time (220 min:30 + 100 + 90), but it has low sensitivity, toxic nucleic acid dyes used, and cannot obtain quantitative results; RTFQ-PCR method has advantages of faster (160 min: 30 + 130) and high sensitivity, however, this assay requires skilled technicians and expensive nucleic acid amplification instrument, which limited the application scope of this method (Table 4).

**TABLE 4:** Parameters comparison of methods used for detection of *Klebsiella pneumoniae* of infants.

Method	Sensitivity	Demands for instrumentation	Potential for specific DNA sequence	Inspection cost	Times
Traditional microbiological method	Low	Medium	No	Medium	2–3 days
PCR-GE	Medium	Medium	No	Medium	220 min (30 + 100+90)^b^
PCR-ICTS^a^	High	Medium	Yes	Low	160 min (30 + 100+30)^c^
SEA-ICTS^a^	High	Low	No	Low	80 min (30 + 20+30)^d^
RTFQ-PCR	High	High	Yes	High	160 min (30 + 130)^e^

^a^ The asterik represents the research methods developed by this study.

^b^ 220 min include the time of DNA extraction for 30 min, PCR for 100 min, electrophoresis for 90 min.

^c^ DNA extraction for 30 min, PCR for 100 min, purification and ICTS for 30 min.

^d^ DNA extraction for 30 min, SEA for 20 min, purification and ICTS for 30 min.

^e^ DNA extraction for 30 min, RTFQ-PCR for 130 min.

In fact, after purification of nucleic acid amplification products (SEA or PCR products), the ICTS detection time only need 2–3 min. In this article, we choose 30 min as a comparative benchmark for the whole process including purification and ICTS. Especially, in our ICTS quantitative testing platform, the SEA-ICTS method was optimized based on PCR-ICTS we established in this study, which is a more rapid, low-costed method compared with PCR-ICTS assay for the detection of *Klebsiella pneumoniae* (KP, *K. pneumoniae*) (KP) in infants. Only needs cheap thermostatic water bath and takes a short detection time, this new method can potentially serve as an efficient POCT method for on-site detection of pathogens and disease outbreaks without fluorescent PCR instruments and professional technicians operation.

In summary, this study aims to eliminate the application of gel electrophoresis in nucleic acid amplification product analysis and replace it with a faster, quantitative and cost-effective ICTS testing platform of SEA-ICTS and PCR-ICTS. The detection sensitivity of these two methods is nearly the same with RTFQ-PCR, but the cost is lower than that of RTFQ-PCR, which is cheaper and more suitable for testing clinical samples, especially in poor working conditions in hospitals. SEA-ICTS and PCR-ICTS methods can be used as alternatives to complement each other, according to the test working conditions. Therefore, due to its high sensitivity, strong specificity, simple operation, low-costed and strong detectability, the new ICTS quantitative testing platform can be used as an ideal tool to quantitatively and accurately determine whether patients, especially infants are infected with *K. pneumoniae*.

## 5 Conclusion

This paper introduces an nFM-based ICTS quantitative testing platform (SEA-ICTS and PCR-ICTS) that can detect *Klebsiella pneumonia* in infants as low as 2.82 × 10^−7^ ng/μL of SEA-ICTS method and 7.7 × 10^−6 ^ng/μL of PCR-ICTS method. The results have showed that the new ICTS methods were fast, non-toxic, simple and low-costed. In particular, the SEA-ICTS method does not require special and expensive equipment and complex technologies to achieve high specificity and sensitivity, facilitate the rapid detection of *K. pneumoniae*, and may become an alternative fast and easy clinical sample detection tool to RTFQ-PCR and the traditional clinical detection methods.

## Data Availability

The original contributions presented in the study are included in the article/Supplementary Material, further inquiries can be directed to the corresponding authors.
